# Supplementary material for: Magnetic skyrmion braids
Source: Nat Commun. 2021 Sep 7;12:5316. doi: 10.1038/s41467-021-25389-7 (PMC8423765; doi:10.1038/s41467-021-25389-7)
Supplement: Supplementary file 1 — Supplementary Information [file 41467_2021_25389_MOESM1_ESM.pdf]

Supplementary Information for "Magnetic skyrmion braids"

This Supplementary Information contains twelve supplementary figures, including experimental and theoretical Lorentz TEM images, phase shift images and images illustrating the three-dimensional structures of skyrmion braids. It also contains a list of references.

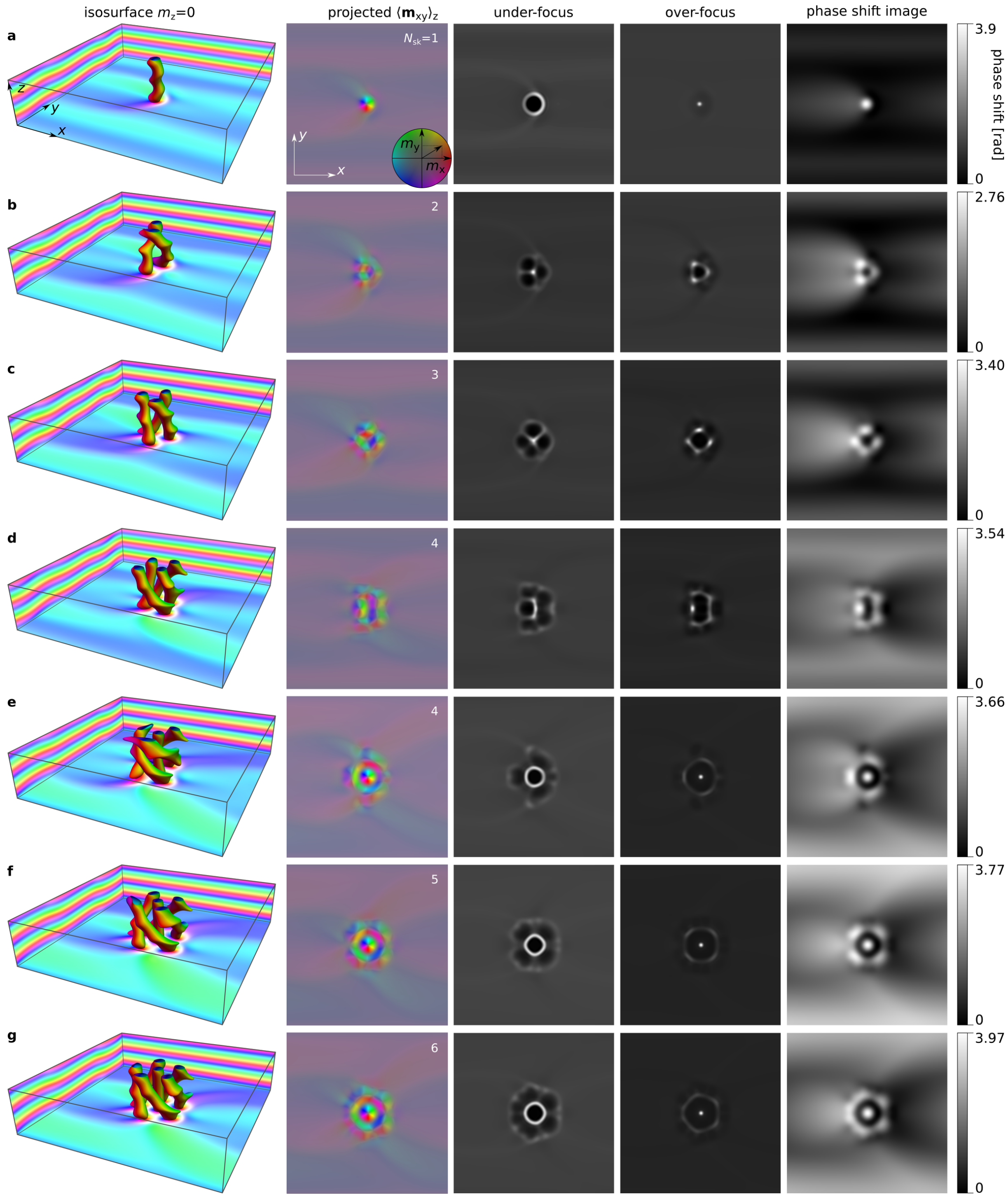

Supplementary Figure 1. Skyrmion braids in an FeGe plate of thickness 180 nm. This figure is an extended version of Fig. 3 in the main text.

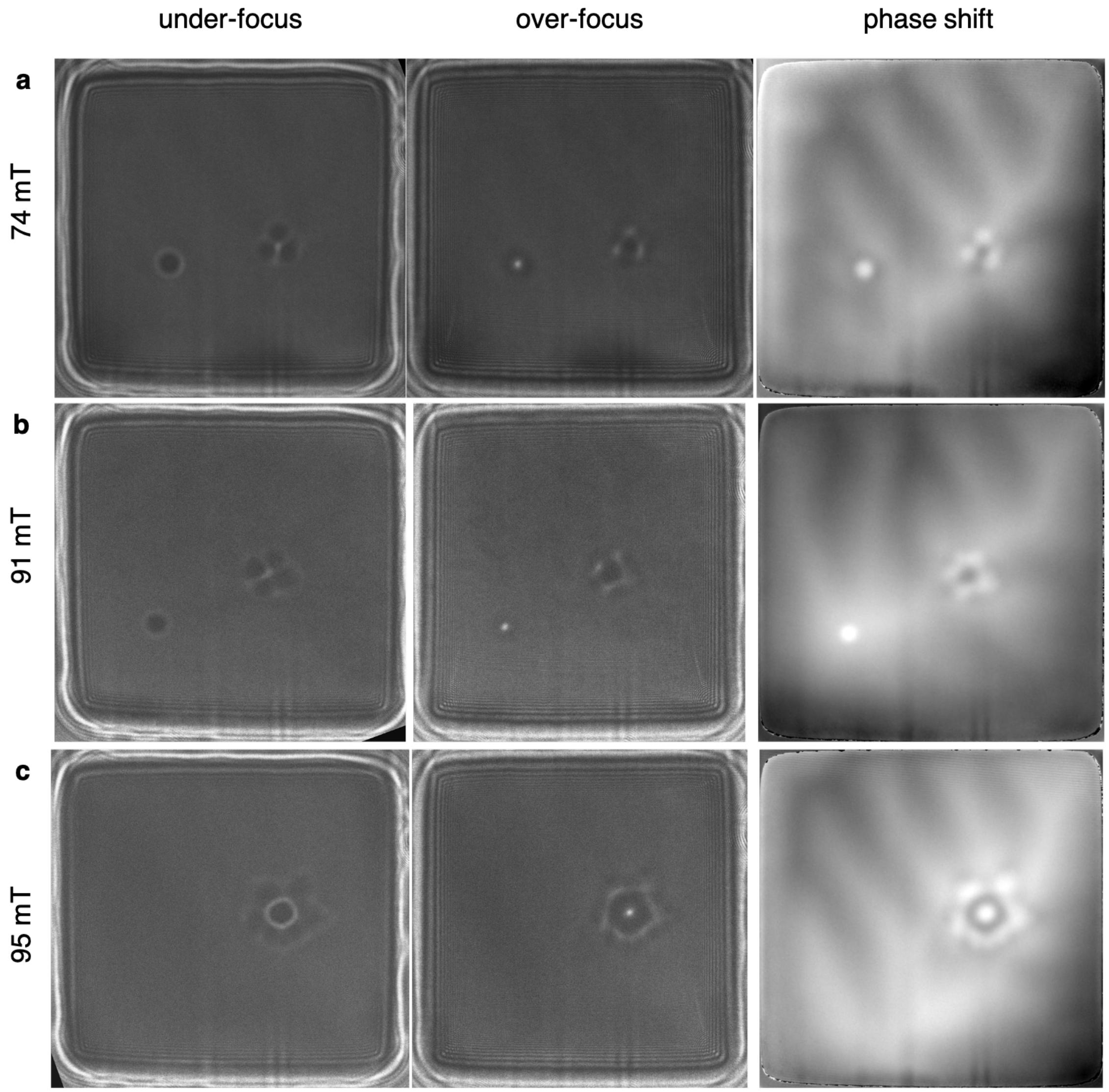

Supplementary Figure 2. Lorentz TEM and phase shift images of skyrmion braids in sample S1. a-c, Skyrmion braids composed of 2, 3 and 6 skyrmion strings, respectively. The applied magnetic fields are labeled on the left of the image. From left to right: under-focus, over-focus Lorentz TEM and phase shift images, respectively. The Lorentz TEM images were recorded at a defocus distance of 400 μm. The specimen temperature was 95 K.

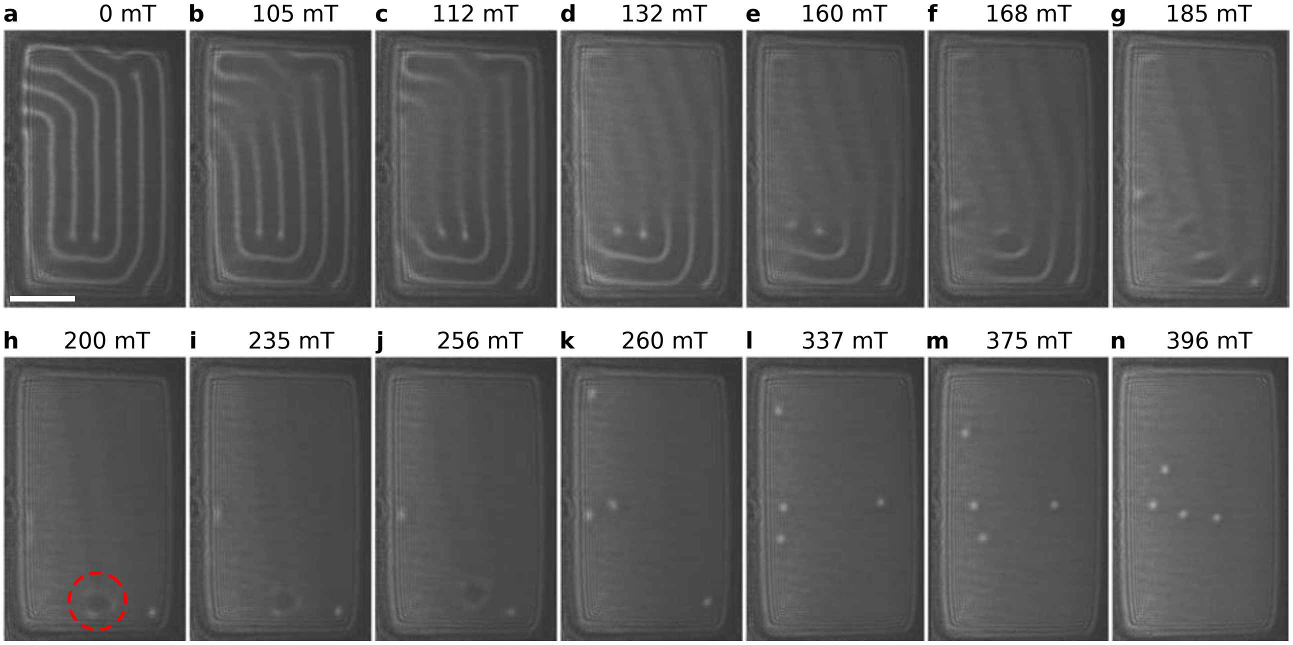

Supplementary Figure 3. Evolution of magnetic states in sample S2 in an increasing applied magnetic field. The Lorentz TEM images show a representative sequence of magnetic states in an increasing out-of-plane applied magnetic field. The magnitude of the applied magnetic field is indicated above each image. The scale bar in **a** is 200 nm. The images were recorded over-focus at a defocus of 400 μm. The specimen temperature was 95 K. Image **a** was recorded in zero field, and shows a helical spin spiral. On increasing the field to 185 mT, the contrast from the helix becomes weaker, as a result of a transition to a surface spin spiral state[1]. In an applied field of $\sim$ 200 mT, an object with an unusually weak ring-like contrast appears, as indicated by a red dashed circle in image **h**. This object was identified to be a skyrmion braid, similar to that depicted in Fig. 3**e** in the main text. Next to the ring-like pattern with a dark inner region (**h**-**j**), two higher-contrast bright spots correspond to straight skyrmion strings, which are attached to the sample edge. On further increasing the applied field to 260 mT, the skyrmion braid untwists into a pair of straight skyrmion strings near the left edge of the sample, as shown in image **k**. The number of skyrmions that appear after the ring-like pattern decays agrees with our theoretical prediction. (See the cluster of two skyrmions in Fig. 3**e** in the main text). Other experimental images of skyrmion braids that are formed from two skyrmion strings in this sample are shown in Extended Data Fig. 4**c** and Figs 5**a** and 5**c**. On further increasing the field, the skyrmions move towards the center of the sample, suggesting saturation of the edge modulations[2]. See also Extended Data Video 3.

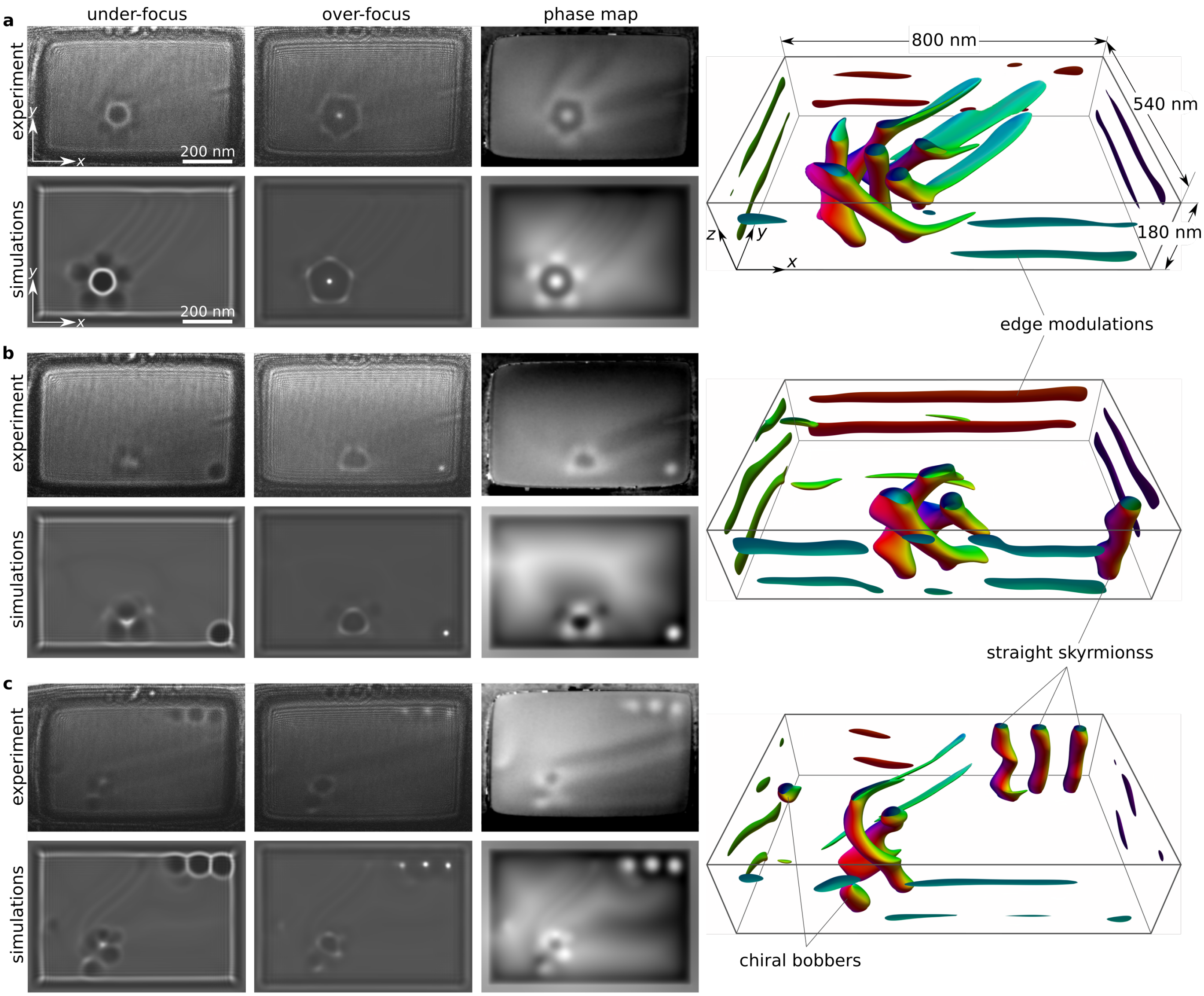

Supplementary Figure 4. Skyrmion braids in a thin plate. This figure is an extended version of Fig. 3 in the main text. The number of skyrmions in **a**, **b** and **c** is 6, 3 and 2, respectively. The right panel shows corresponding calculated three-dimensional magnetization distributions obtained from micromagnetic simulations. An important feature of the states in **b** and **c** is the coexistence of skyrmion braids and skyrmion strings. The state in **c** includes a pair of chiral bobbers. This magnetization distribution provides the best agreement that we found between experimental and theoretical images.

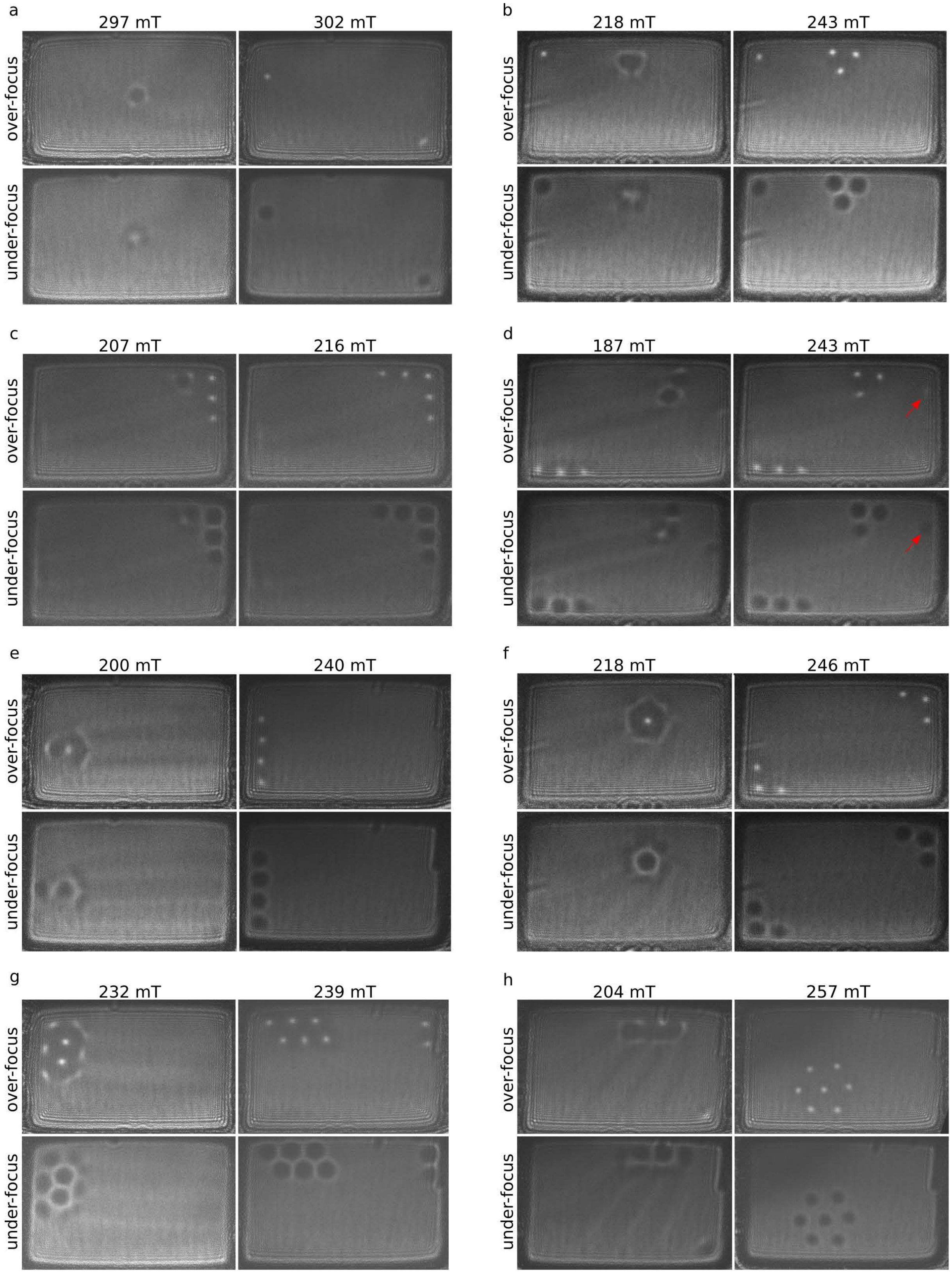

Supplementary Figure 5. Lorentz TEM images of skyrmion braids with different numbers of skyrmion strings in sample S2. The number of skyrmion strings in each skyrmion braid is 2 in **a**, 3 in **b**, 2 in **c**, 3 in **d**, 4 in **e**, 6 in **f**, 8 in **g** and 7 in **h**. The red arrow in **d** indicates the position of a chiral bobber. Images **a-h** each show over-focus and under-focus Lorentz TEM images of skyrmion braids before (left) and after (right) transitions into straight skyrmion strings by increasing the applied magnetic field. The magnitude of the applied field is indicated above each image. The defocus was 400 μm. The specimen temperature was 95 K.

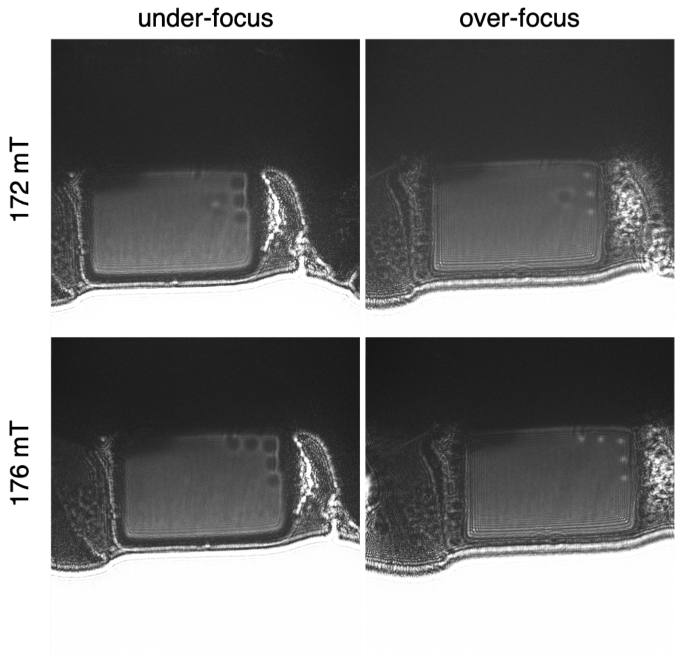

Supplementary Figure 6. Lorentz TEM images of skyrmion braids at 170 K in sample S2. The skyrmion braids are composed of two skyrmion strings. Under-focus and over-focus Lorentz TEM images are shown before (top) and after (bottom) a transition to two straight skyrmion strings. Three additional skyrmion strings are attached to the sample edge. The applied magnetic field is indicated on the left of each image. The images were recorded at a defocus of 400 μm.

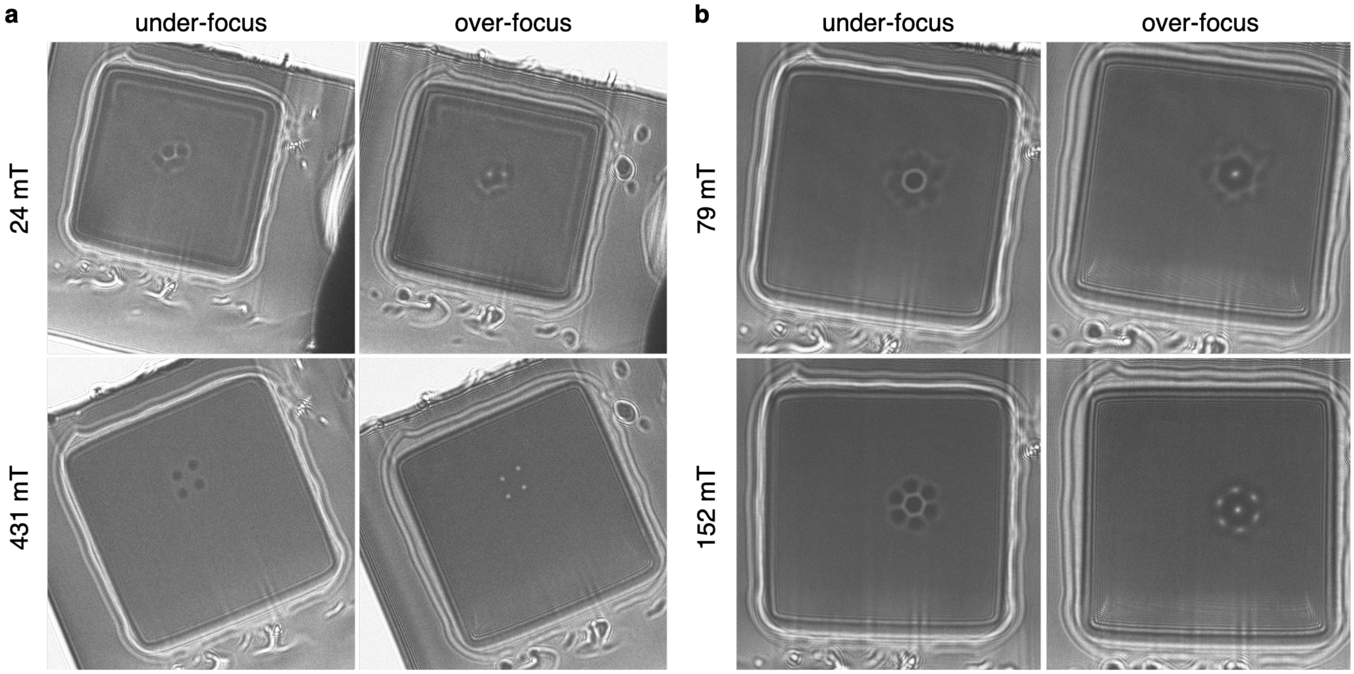

Supplementary Figure 7. Lorentz TEM images of skyrmion braids composed of different numbers of skyrmion strings in sample S1. The number of skyrmions in each skyrmion braid is 4 in **a** and 7 in **b**. Under-focus and over-focus Lorentz TEM images are shown before (top) and after (bottom) a transition to straight skyrmion strings by increasing the applied magnetic field. The applied magnetic field is indicated on the left of each image. The images were recorded at a defocus of 400 μm. The specimen temperature was 95 K.

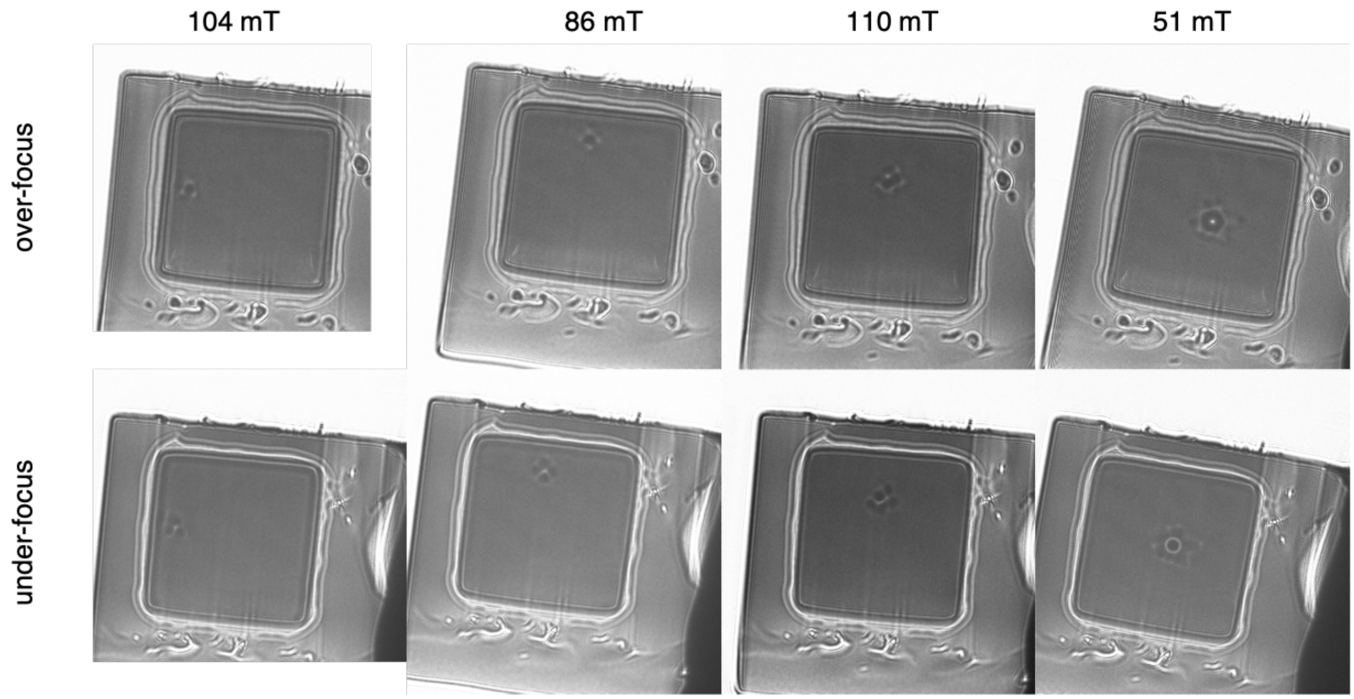

Supplementary Figure 8. Lorentz TEM images of skyrmion braids at 120 K in sample S1. Each column shows over-focus and under-focus Lorentz TEM images. From left to right, the skyrmion braids contain 2, 3, 4 and 6 skyrmion strings, respectively. The applied magnetic field is indicated above each image. The images were recorded at a defocus of 400 μm.

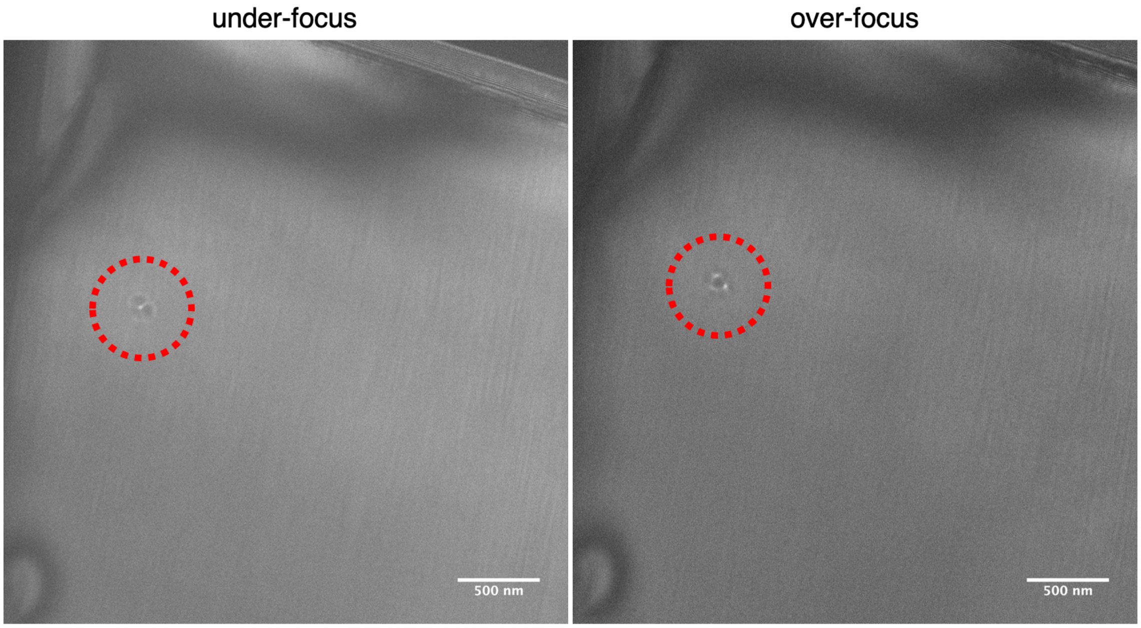

Supplementary Figure 9. Lorentz TEM images of a skyrmion braid in the large sample S3. The braid is composed of two skyrmions. The applied magnetic field is 45 mT. The images were recorded at a defocus of 400 μm. The specimen temperature was 95 K.

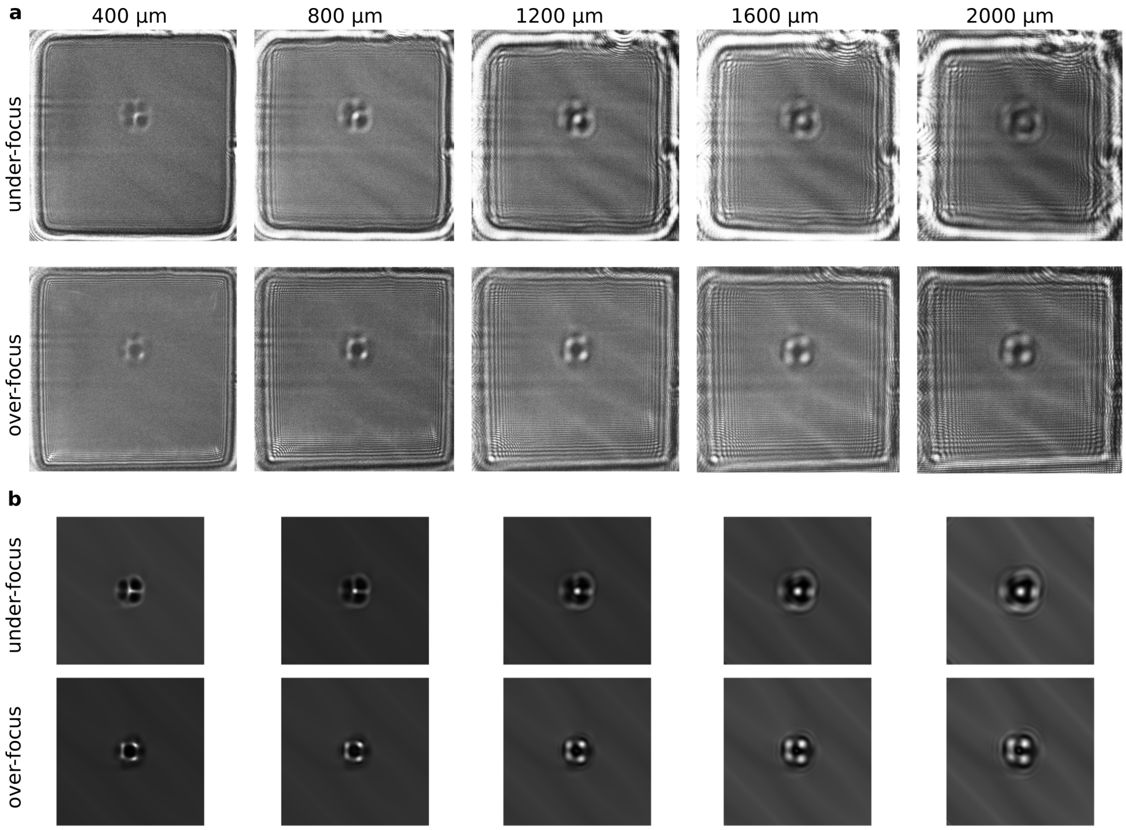

Supplementary Figure 10. Lorentz TEM images of skyrmion braids as a function of defocus. **a** Experimental Lorentz TEM images of a skyrmion braid composed of three skyrmion strings in sample S1 recordd in an applied magnetic field of 62 mT at a specimen temperature of 95 K. **b** Theoretical Lorentz TEM images calculated for the skyrmion braid in Fig. 3 **f** in the main text. The defocus is indicated above each image.

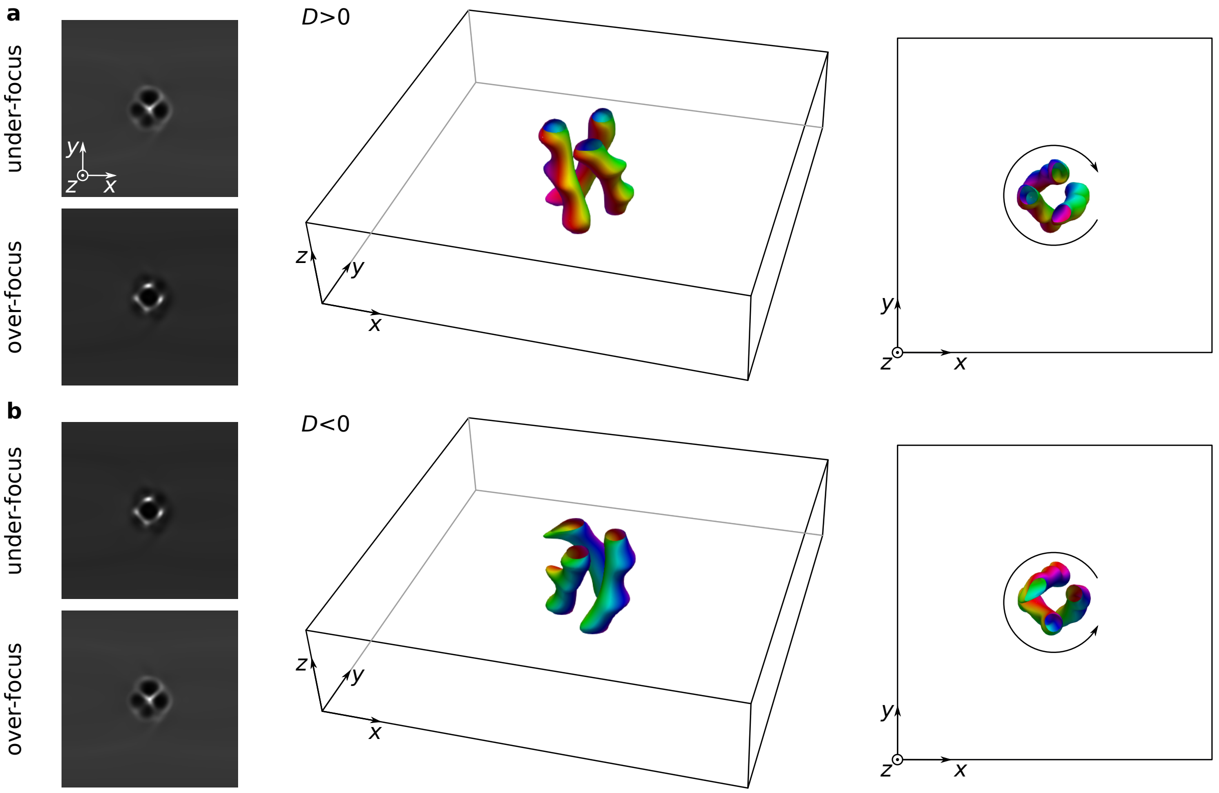

Supplementary Figure 11. Skyrmion braids in crystals of opposite chirality. **a** and **b**, Skyrmion braid composed of three skyrmion strings calculated for the same conditions as in Fig. 3 in the main text, but for DMI constants of opposite sign. The absolute value of the DMI constant and other material parameters are given in the Methods section. The panels contain simulated over-focus and under-focus Lorentz TEM images for a defocus of 400 μm (left) and isosurfaces for corresponding skyrmion braids (right). The contrast of the the over-focus and under-focus Lorentz TEM images is interchanged when the DMI constant changes sign.

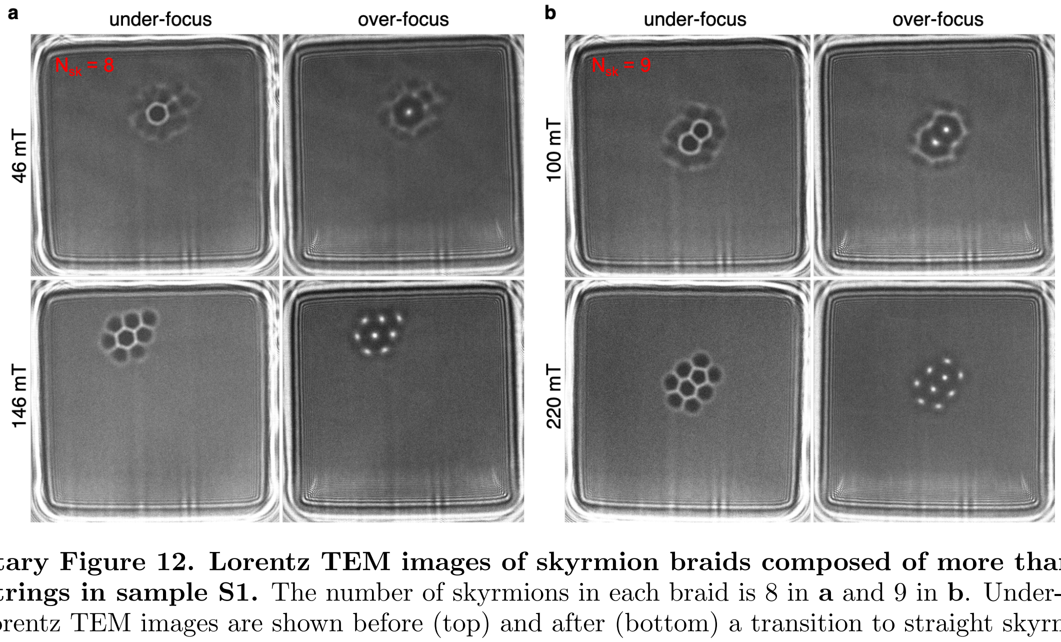

Supplementary Figure 12. Lorentz TEM images of skyrmion braids composed of more than 7 skyrmion strings in sample S1. The number of skyrmions in each braid is 8 in **a** and 9 in **b**. Under-focus and over-focus Lorentz TEM images are shown before (top) and after (bottom) a transition to straight skyrmion strings by increasing the applied magnetic field. The applied magnetic field is indicated on the left of each image. The images were recorded at a defocus of 400 μm. The specimen temperature was 95 K.

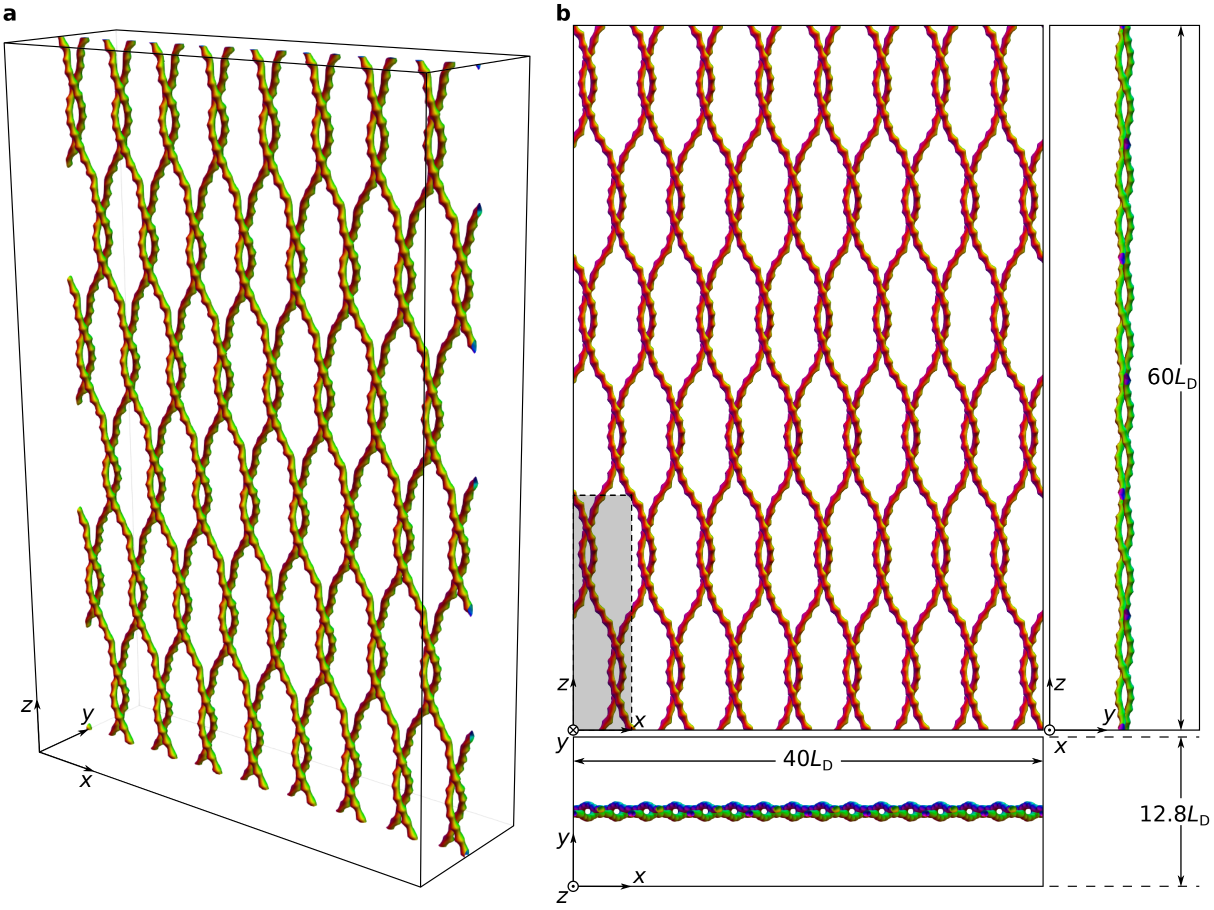

Supplementary Figure 13. "Chicken wire" superstructure braided from skyrmion strings. a Perspective projection. **b** Side views with the domain size indicated. This texture was obtained by minimizing the energy of the unit cell (shaded rectangle) from a suitable initial guess for periodic boundary conditions in an applied field of $B_{\mathrm{ext}} = 0.392 B_{\mathrm{c}}$. The unit cell size is not assumed to be optimal and was chosen to be 5 $L_{\mathrm{D}} \times 20$ L_{D} in the xz plane.

[1] Rybakov, F. N., Borisov, A. B., Blügel, S. & Kiselev, N. S. New spiral states and skyrmion lattice in 3D model of chiral magnet. *New J. Phys.* **18**, 045002 (2016).
[2] Du, H. et al. Interaction of individual skyrmions in a nanostructured cubic chiral magnet. *Phys. Rev. Lett.* **120**, 197203 (2018).
