# Supplementary material for: Magnetic skyrmion braids
Source: Nat Commun. 2021 Sep 7;12:5316. doi: 10.1038/s41467-021-25389-7 (PMC8423765; doi:10.1038/s41467-021-25389-7)
Supplement: Supplementary file 9 — Description of Additional Supplementary Files [file 41467_2021_25389_MOESM9_ESM.pdf]

Title: Supplementary Movie 1:
Description: Real-time energy minimization, showing braiding in a cluster of six skyrmions. Skyrmion strings spontaneously twist when the applied magnetic field is decreased. The film thickness is 180 nm.

Title: Supplementary Movie 2:
Description: Crafting of magnetic textures inside a plate, with simultaneous energy relaxation.

Title: Supplementary Movie 3:
Description: Lorentz over-focus images for sample S2. The movie shows the evolution of magnetic states with the increase of the external magnetic field.

Title: Supplementary Movie 4:
Description: Lorentz over-focus images for sample S2. The movie illustrates the reproducibility of skyrmion braiding with increasing and decreasing field.

Title: Supplementary Movie 5:
Description: Real-time Lorentz TEM contrast recorded over-focus, illustrating the protocol for skyrmion braid nucleation.

Title: Supplementary Movie 6:
Description: Real-time Lorentz TEM observation of skyrmion braiding with increasing and decreasing applied magnetic field recorded over-focus and under-focus for a skyrmion braid composed of 6 skyrmion strings. The movie illustrates the reproducibility of skyrmion braiding in sample S1.
